# Metagenomic survey of the taxonomic and functional microbial communities of seawater and sea ice from the Canadian Arctic

**DOI:** 10.1038/srep42242

**Published:** 2017-02-08

**Authors:** Etienne Yergeau, Christine Michel, Julien Tremblay, Andrea Niemi, Thomas L. King, Joanne Wyglinski, Kenneth Lee, Charles W. Greer

**Affiliations:** 1 Centre INRS-Institut Armand-Frappier, Institut National de la Recherche Scientifique, Université du Québec, Laval, Québec, Canada; 2 Fisheries and Oceans Canada, Winnipeg, Manitoba, Canada; 3 National Research Council Canada, Energy, Mining and Environment, Montréal, Québec, Canada; 4 Fisheries and Oceans Canada, Dartmouth, Nova Scotia, Canada; 5 Commonwealth Scientific Industrial Research Organization, Kensington, Western Australia, Australia

## Abstract

Climate change has resulted in an accelerated decline of Arctic sea ice since 2001 resulting in primary production increases and prolongation of the ice-free season within the Northwest Passage. The taxonomic and functional microbial community composition of the seawater and sea ice of the Canadian Arctic is not very well known. Bacterial communities from the bottom layer of sea ice cores and surface water from 23 locations around Cornwallis Island, NU, Canada, were extensively screened. The bacterial 16S rRNA gene was sequenced for all samples while shotgun metagenomics was performed on selected samples. Bacterial community composition showed large variation throughout the sampling area both for sea ice and seawater. Seawater and sea ice samples harbored significantly distinct microbial communities, both at different taxonomic levels and at the functional level. A key difference between the two sample types was the dominance of algae in sea ice samples, as visualized by the higher relative abundance of algae and photosynthesis-related genes in the metagenomic datasets and the higher chl *a* concentrations. The relative abundance of various OTUs and functional genes were significantly correlated with multiple environmental parameters, highlighting many potential environmental drivers and ecological strategies.

The Canadian Arctic Archipelago constitutes an extensive Arctic continental shelf area, of socio-economic and ecological importance due to its highly productive ice-associated ecosystem, its role as a refuge for multiyear ice, and as a northern navigation route via the Northwest Passage^1^,^2^,^3^. Climate change has resulted in an accelerated decline of Arctic sea ice since 2001 and revised predictions suggest a near complete loss of Arctic summer sea ice by mid-century, or earlier^1^,^4^. The effects of loss of sea ice on marine ecosystems are numerous and include primary production increases^5^,^6^ and changes in microbial communities^7^,^8^. Another major concern associated with the reduction of Arctic sea ice is the increased risk of an accidental oil spill in this fragile and pristine environment due to the prolongation of the ice-free season for marine traffic within the Northwest Passage.

However, the microbial community composition and genomic content of seawater and sea ice around the Northwest Passage, or in the Arctic in general, is poorly understood. Metagenomic studies conducted in the marine environment in the Canadian high Arctic are still limited^9^, but some interesting information was obtained through other methods or in other similar environments. For instance, *Gammaproteobacteria, Alphaproteobacteria* and *Flavobacteria* were observed to be common to both sea ice and seawater^10^. Recent studies revealed similar diversity in the ice and surface waters at two locations of the Canadian Arctic^11^,^12^. In the Laptev Sea, a clone library study showed the importance of physico-chemical forcings, such as temperature and riverine input, in shaping surface water bacterial communities^13^. Bacterial diversity at the subsurface chlorophyll *a* maximum, obtained from SSU rRNA gene amplicon sequencing, was also found to be influenced by bio-physical forcings, in particular complex organic matter content^8^.

The objectives of the present survey were to 1) characterize bacterial community structures in seawater and the immediate bottom layer of sea ice cores (referred to as sea ice) recovered near Cornwallis Island in the Northwest Passage, and 2) relate the relative abundance of key taxa and functional genes to environmental parameters. In order to attain these goals, the 16S rRNA gene was amplified and sequenced from sea ice and seawater samples taken at 23 locations around Cornwallis Island in the Canadian high Arctic, while 16 selected samples (6 sea ice and 10 seawater samples) were also subjected to shotgun metagenomic sequencing.

## Material and Methods

### Sample collection

**Figure 1:**
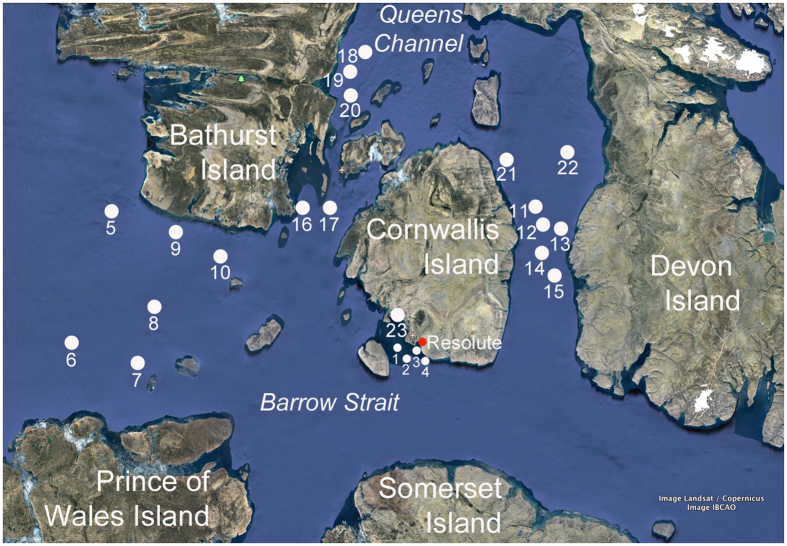
Location of sampling stations in the Canadian Arctic Archipelago (Nunavut). The map was generated using Google Earth (version 7.1; https://www.google.com/earth/). Map data © Landsat/Copernicus/IBCAO.

Samples of surface water and ice bottom samples were collected at 23 stations surrounding Cornwallis Island, in Lancaster Sound, Wellington Channel and adjacent channels, between 74.10′ and 75.93′N and 92.64′ and 101.34′W (Fig. 1). The sampling was carried out between May 4 and 18, 2011, which corresponded to the period of the ice algal bloom. At each station, seven or eight ice cores were collected using a 9 cm diameter manually-driven ice corer (Mark II coring system, Kovacs Enterprises, Lebanon, NH, USA). The bottom 3 cm section of each core was cut using a clean stainless steel saw and brought back to the shore laboratory for analysis (core volume ≅ 190 ml). Three cores, used for biomass measurements and DNA analyses, were pooled together and melted with the addition of 0.2 μm sterile-filtered surface seawater collected at the time of sampling (referred to as “sea ice” samples). This procedure was performed to dilute the thick microbial mats found at the bottom of the cores and maintain osmotic pressure in the samples to be filtered for DNA extraction. The three other cores, used for chemical analyses, were placed in a sterile bag and melted without the addition of filtered seawater. Sterile gloves were always worn when manipulating the cores. All cores were slowly melted at 4 °C in the dark. Surface water (the layer of water directly under ice) was collected using a submersible pump mounted on an articulated arm deployed at the sea ice-seawater interface (referred to as “seawater” samples). At the time of sampling, snow thickness and ice thickness were measured at 5 sites near core collection.

### Sample processing and analyses

Sea ice and seawater samples were analyzed for chlorophyll *a* (chl *a*), bacterial abundance, particulate organic carbon (POC), dissolved organic carbon (DOC), dissolved nitrogen (DN), macro-nutrients (NO_3_+NO_2_, PO_4_, SiOH_4_) and salinity. Chl *a* fluorescence was read on a 10AU Turner Design fluorometer calibrated using pure chl *a* extract (Sigma-Aldrich, Oakville, ON, Canada) and concentrations were calculated according to Parsons *et al*.^14^. Bacterial abundance was measured by flow cytometry as detailed in Belzile *et al*.^15^. POC was measured on a Carlo Erba NC2500 elemental analyzer (Carlo Erba Reagents, Val de Reuil, France). Dissolved organic carbon and nitrogen were measured using a Shimadzu TOC-VCHP analyzer with a TNM-1 Total Nitrogen module (Shimadzu Corp., Kyoto, Japan). Analyses were systematically checked against deep Sargasso Sea reference water from Hansell’s Certified Reference Materials (University of Miami, Miami, FL, USA). Samples for nutrient analysis were frozen at −80 °C and later analyzed on a SmartChem 200 chemistry analyzer (Unity Scientific, Brookfield, CT, USA). Additional details on the methods can be found in Michel and Niemi^16^.

### DNA extraction

DNA extractions were performed on duplicate samples of the melted sea ice (50–100 ml) or seawater samples (1000 ml) filtered on 0.22 μm nitrocellulose filters (Millipore, Billerica, MA, USA). The filters were immediately frozen and stored at −80 °C until DNA and RNA extraction. The Powerwater RNA Isolation Kit (MoBio Laboratories, Carlsbad, CA, USA) was used for seawater nucleic acid extraction of the filters according to the manufacturer’s protocol, modified for the presence of lysis-resistant organisms and the omission of DNAse treatment for total nucleic acid extraction. The protocol for DNA extraction of the sea ice filters was based on a modified CTAB (cetyltrimethylammonium bromide) method of Ausubel *et al*.^17^ to address the large amounts of exopolymeric substances found on the sea ice filters. Volumes of reagents were tripled in comparison to the original protocol to ensure full submersion of filters. DNA was quantified by the Picogreen method (Life Technologies, Burlington, On, Canada) on a Tecan Magellan Fluorimeter (Tecan Group Ltd., Männedorf, Switzerland). DNA extracts were stored at −80 °C until further analysis.

### Amplification and sequencing of the bacterial 16S rRNA gene

Libraries for sequencing were prepared according to Illumina’s “16S Metagenomic Sequencing Library Preparation” guide (Part # 15044223 Rev. B), with the exception of using Qiagen HotStar MasterMix for the first PCR (“amplicon PCR”) and halving reagent volumes for the second PCR (“index PCR”). The template specific primers were (without the overhang adapter sequence): 515f (5′-GTGCCAGCMGCCGCGGTAA-3′) and 806R (5′-GGACTACHVGGGTWTCTAAT-3′). The first PCR (“amplicon PCR”) was carried out for 25 cycles with annealing temperatures of 55 °C. Diluted pooled samples were loaded on an Illumina MiSeq and sequenced using a 500-cycle MiSeq Reagent Kit v3.

### Metagenomic sequencing

Following UPGMA cluster analysis of the samples based on the 16S rRNA gene dataset (not shown), representative samples of major clusters were selected for metagenomic sequencing. Each DNA library was prepared for sequencing using the Ion Xpress Plus Fragment Library Kit (Life Technologies) with the Ion Xpress Barcode Adapters 1–16 (Life Technologies), using the Ion Shear Plus Reagents. Size selection was performed using a Pippin Prep instrument (SAGE Science, Beverly, MA) set to “tight” collection with a base pair target setting of 315 to collect fragments with a median of 200–300 bp as recommended in the “Ion Express Plus gDNA Fragment Library Preparation” manual (Life Technologies publication number 4471989, revision L). Barcoded libraries were pooled in an equimolar ratio three by three. A total of 3.50 × 10^7^ molecules were used in an emulsion PCR using the Ion Xpress 200 Template Kit (Life Technologies) as described in Sanschagrin and Yergeau^18^. Sequencing of the pooled libraries was performed using the PGM system with the Ion Sequencing 200 kit and 316 chips.

### 16S rRNA gene sequence data analysis

Sequences were analyzed through our internal rRNA short amplicon analysis pipeline as previously described^19^,^20^. Briefly, reads were filtered, assembled with their overlapping paired-end and clustered at 97% identity. Taxonomy was then assigned to each cluster based on the Greengenes taxonomy (Greengenes v13_5) and OTU tables were generated, filtered to exclude eukaryotes and chloroplasts and normalized as described in McMurdie and Holmes^21^. This normalized OTU table was used for downstream analysis and for computing alpha and beta diversity metrics. Sequencing statistics are presented in Supplementary Table S1.

### Shotgun metagenomic sequence data analysis

Shotgun metagenomic sequences were submitted to MG-RAST 3.0^22^ where they were de-replicated using the method of Gomez-Alvarez *et al*.^23^ and trimmed using the dynamic trimming method of Cox *et al*.^24^ in a way that each individual sequence would contain a maximum of 5 bases below a Phred score of 15. Within MG-RAST, significant matches were defined as having 60% sequence identity over at least 15 amino acids or 50 bp and with an e-value below 10^−5^. MG-RAST classifies sequences using the “subsystems technology”, in which each sequence is assigned to a manually curated subsystem^25^. The subsystems are grouped in categories in a hierarchical fashion, ranging from “Functions” (most detailed category, e.g. “Photosystem II CP47 protein (PsbB)”) to “Level1” (least detailed category, e.g. “Photosynthesis”), with intermediate categories Level 2 (e.g. “Electron transport and photophosphorylation”) and Level 3 (e.g. “Photosystem II”).

### Statistical analyses

All statistical analyses were carried out in R (The R foundation for Statistical Computing, Vienna, Austria). Paired and non-paired t-tests were performed using the t.test function (or the non-parametric wilcox.test if needed), Spearman rank-order correlations (r_s_) using the “cor.test” function, dissimilarity calculation using the vegdist function of the vegan library, principal coordinate analyses (PCoA) using the “cmdscale” function and Permanova using the adonis function of the vegan library. Unweighted Pair Group Method with Arithmetic Mean (UPGMA) was carried out based on the UniFrac distance matrices using the “agnes” function of the vegan library.

### Data deposition

Raw sequence data produced in this study was deposited in NCBI under the BioProject accession PRJNA266338 and the SRA accession SRP051064. Annotated metagenomes are available in MG-RAST under IDs 4504402.3–4504417.3 in project 2397.

## Results

### Biochemical characteristics

A summary of the biochemical characteristics of sea ice and seawater is presented in Table 1, showing significantly higher concentrations of chl *a*, particulate organic carbon, dissolved organic carbon and nitrogen, bacterial abundances, and nutrient (N and P) concentrations in the sea ice compared to seawater (t-tests, p < 0.05). In contrast, Si(OH)_4_ and salinity were significantly higher in the seawater as compared to the sea ice (t-tests, p < 0.001).

### Taxonomic community profile

**Figure 2:**
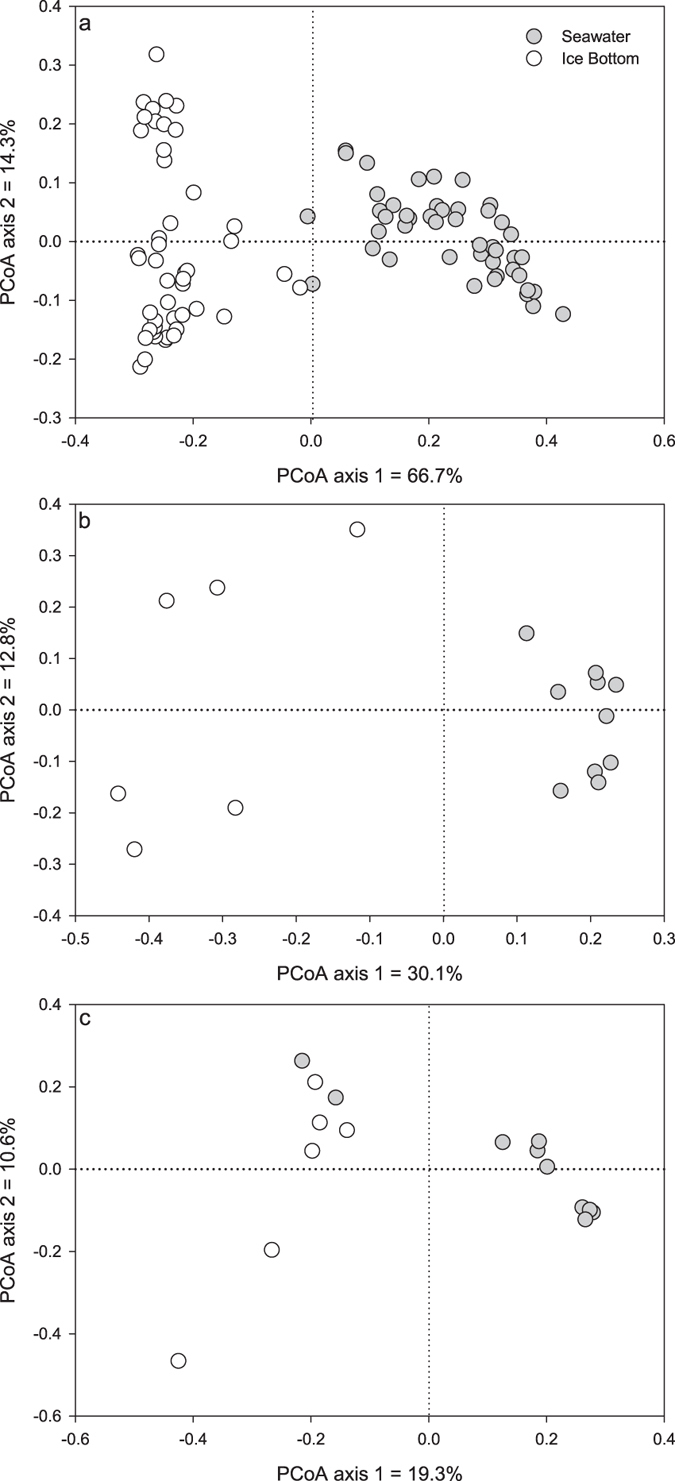
Principal coordinate ordinations of (**a**) UniFrac distances calculated from 16S rRNA gene, (**b**) Bray-Curtis distances calculated from relative abundance of species in metagenomic datasets and c) Bray-Curtis distances calculated from relative abundance of functions in metagenomic datasets.

Not surprisingly, when plotting principal coordinates calculated from UniFrac distances (based on 16S rRNA gene sequences) a significant difference (Permanova tests: F = 146.66, P < 0.001) was observed between the microbial communities in seawater and sea ice samples (Fig. 2a). The microbial communities associated with the sea ice were more spatially variable than the microbial communities in seawater (average weighted UniFrac distance between samples of 0.253 for sea ice vs. 0.233 for seawater) (Fig. 2a). Seawater samples were significantly richer (Chao 1 richness: 128.1 for sea ice and 322.1 for seawater, t = 17.4, P < 0.001) and more diverse than sea ice samples (Inverse Simpson diversity index: 0.869 for sea ice and 0.963 for seawater; t = 7.7, P < 0.001).

Similar differences between the sea ice and seawater samples were visible in the metagenomic dataset, both for the species-level taxonomic table (Fig. 2b) and the MG-RAST “Function” level subsystem table (Fig. 2c). Although the differences between sea ice and seawater samples were visually less clear for the function-level ordination (Fig. 2c), Permanova tests revealed that there were significant differences between seawater and sea ice samples for both the taxonomic and function tables (F = 11.49, P < 0.001 and F = 3.55, P < 0.001, respectively). The seawater samples showed less variability than the sea ice for both the taxonomic and function tables (average Bray-Curtis distance between samples of 0.491 and 0.700 for sea ice vs. 0.307 and 0.460 for seawater, respectively) (Fig. 2b,c).

**Figure 3:**
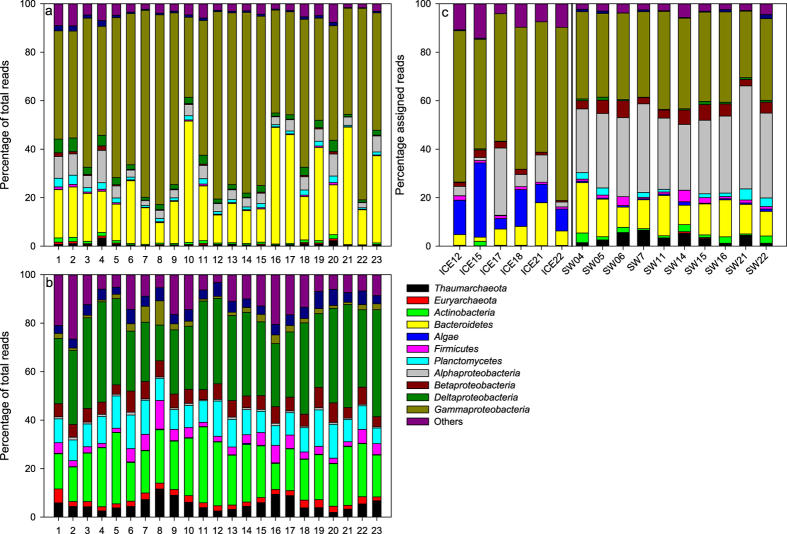
Community composition for each station at the phylum/class level for (**a**) sea ice based on 16S rRNA gene datasets, (**b**) seawater based on 16S rRNA gene datasets and (**c**) sea ice and seawater based on metagenomic datasets. The numbers on the x-axis represent the sampling station number as shown in Fig. 1. The “Others” category comprised 31 low-abundance phyla/classes.

The differences observed between sea ice and seawater samples were also visible at relatively high taxonomic levels (phylum-class) (Fig. 3). Seawater samples were significantly enriched in *Thaumarcheota, Euryarchaeota, Actinobacteria, Cyanobacteria, Fimicutes, Planctomycetes, Betaproteobacteria, Deltaproteobacteria*, and *Verrucomicrobia* and significantly depleted in *Bacteroidetes, Alphaproteobacteria* and *Gammaproteobacteria* when compared to sea ice samples. Some of these trends were also visible in the metagenomic datasets (Fig. 3c) with significantly higher relative abundance of *Thaumarchaeota* (t = 5.43, P < 0.001), *Actinobacteria* (t = 2.92, P < 0.05), *Alphaproteobacteria* (t = 5.41, P < 0.01), *Betaproteobacteria* (t = 4.38, P < 0.001), *Deltaproteobacteria* (t = 3.91, P < 0.01), *Planctomycetes* (t = 4.17, P < 0.01) and *Verrucomicrobia* (t = 2.91, P < 0.05) in the seawater as compared to sea ice and a significantly lower relative abundance of diatoms (t = 3.82, P < 0.05) and *Gammaproteobacteria* (t = 5.70, P < 0.01) in the seawater as compared to sea ice. As expected in view of the biases associated with PCR amplification, the relative abundance of some phyla varied considerably between 16S rRNA gene and metagenomic datasets (Fig. 3).

**Figure 4:**
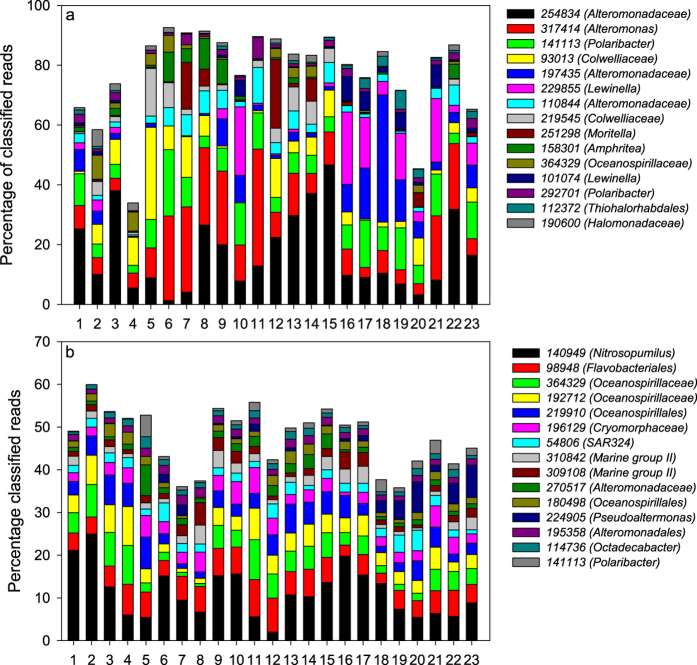
Relative abundance of the 15 most abundant OTUs in the 16S rRNA gene datasets for (**a**) sea ice samples and (**b**) seawater samples. The numbers on the x-axis represent the sampling station number as shown in Fig. 1.

In the 16S rRNA gene datasets, the sea ice was dominated by OTUs related to *Alteromonadaceae, Polaribacter*, and *Colwelliaceae* (Fig. 4a), while the seawater was dominated by OTUs related to *Nitrosopumilus, Flavobacteriales* and *Oceanospirillaceae* (Fig. 4b). The 15 most abundant OTUs depicted in Fig. 4 accounted in most cases for more than 80% of the OTUs for the sea ice, but less than 60% of the OTUs for the seawater (Fig. 4), in line with the higher diversity observed in sea water.

### Functional community profile

**Figure 5:**
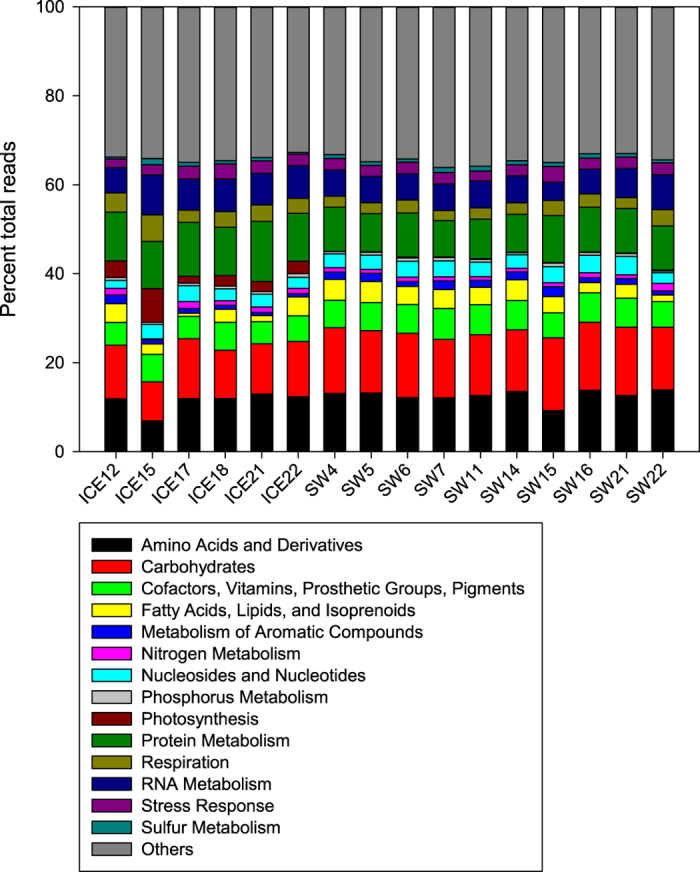
Relative abundance of the MG-RAST “Level 1” functional categories based on shotgun metagenomic datasets for sea ice and seawater. The “Others” category comprised 14 other low-abundance MG-RAST “Level 1” functional categories.

As mentioned above, there were significant differences between the functional profiles of seawater and sea ice based on the relative abundance of MG-RAST “Functions” (Fig. 2d). These differences were particularly visible in photosynthesis-related genes (Fig. 5), with significantly more genes related to this category in the sea ice samples (U = 60, P = 0.000250). Many other gene categories (MG-RAST “Level 1”) had significantly higher relative abundance in the sea ice: Motility and Chemotaxis (t = 4.30, P = 0.00107), Protein Metabolism (t = 8.16, P = 0.00523), Respiration (t = 2.77, P = 0.0269) and RNA Metabolism (t = 2.38, P = 0.0395) (Fig. 5). In contrast, some other gene categories showed significantly higher relative abundance in sea water: Amino Acids and Derivatives (U = 11, P = 0.0420), Carbohydrates (t = 4.23, P = 0.00366), Cofactors, Vitamins, Prosthetic Groups, Pigment (t = 2.85, P = 0.0215) and Dormancy and Sporulation (t = 3.71, P = 0.00236) (Fig. 5).

**Figure 6:**
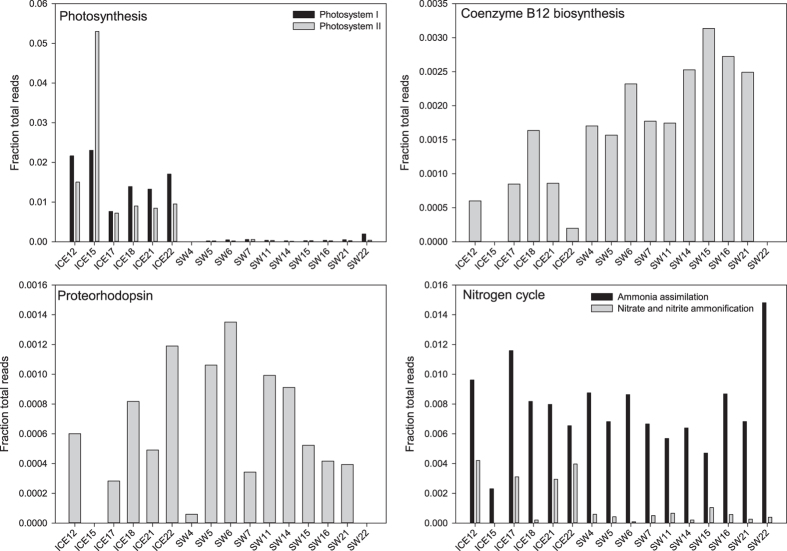
Relative abundance of the MG-RAST “Level 2” and “Level 3” functional categories based on shotgun metagenomic datasets for sea ice and seawater.

The metagenomic datasets were also compared at a finer level (MG-RAST “Level 2” and “Level 3”). There were 160 “Level 2” categories and 988 “Level 3” categories, therefore only categories having a direct link to our data are presented in Fig. 6. The relative abundance of sequences related to “Coenzyme B12 biosynthesis” was significantly higher in the seawater as compared to the sea ice (t = 3.61, P = 0.00295), while the sequences related to both photosystem I and photosystem II had significantly higher relative abundance in sea ice as compared to sea water (Wilcox W = 60 and P = 0.000250 for both) (Fig. 6). The relative abundance of genes related to “Proteorhodopsin”, “Ammonia assimilation” and “Nitrate and nitrite ammonification” was variable between sampling sites and was not significantly different between seawater and sea ice (Fig. 6).

### Environmental drivers

Correlation and canonical correspondence analyses (CCA) were used to identify linkages between OTUs, functional genes and measured environmental variables/constituents (described in Table 1). Given the expected significant differences between sea ice and seawater constituents, the two sample types were treated separately during the analyses. The effect of environmental variables on the microbial community structure was tested by CCA with forward selection of variables to be included in the model. For sea ice 16S rRNA gene datasets, salinity, total bacteria abundance, depth and POC were selected in the model. For the seawater 16S rRNA gene datasets, total N and Si(OH)_4_ were selected. For the MG-RAST “Function” dataset, NO_2_+NO_3_ was selected for the sea ice, while no variable was significant for the seawater.

Many OTUs showed significant correlations with environmental variables. In the sea ice 16S rRNA gene datasets, 461 Spearman correlations were significant (P < 0.05), with 387 being negative and 74 being positive. Salinity was the factor most often positively correlated with OTUs (15 times), while POC was the factor most often negatively correlated with OTUs (71 times). In the seawater 16S rRNA gene dataset, 392 Spearman correlations were significant (175 positive, 217 negative). Si(OH)_4_ was the factor having the highest number of positive (23) and negative (37) correlations. For the sea ice MG-RAST “Function” dataset, 499 correlations were significant (94 positive and 405 negative), with total bacteria showing the highest number of positive correlations with functions (26) and NO2+NO3 showing the highest number of negative correlations (68). For the seawater MG-RAST “Function” dataset, 1627 correlations were significant (659 positive and 968 negative), with DOC showing the highest number of positive correlations (149) with functions and POC showing the highest number of negative correlations (235). The complete list of significant (P < 0.05) correlations is presented in Supplementary Table S2.

## Discussion

In the present study, we compared seawater and the immediately overlying sea ice at 23 locations around Cornwallis Island in the Canadian high Arctic. It is one of the first studies to simultaneously look at linked seawater and sea ice samples using shotgun metagenomics and 16S rRNA gene sequencing and to relate this genomic data to potential environmental drivers. As expected, seawater and sea ice exhibited large differences in terms of chemistry, community composition and functional gene content. A key difference between the two sample types was the dominance of algae in sea ice samples, as visualized by the higher relative abundance of algae and photosynthesis-related genes in the shotgun metagenomic datasets and the high sea ice chl *a* concentrations. Metagenomic datasets from this study showed that these algae were mainly diatoms, confirming visual observations during sampling and corresponding with ice algal taxonomic studies near the study area^26^. The high biomass of primary producers in the sea ice is likely a key/primary driver of the observed differences in microbial communities and nutrient availability^27^. Indeed, primary producers, such as algae, are responsible for the increased availability of labile carbon^28^, benefiting heterotrophs, such as *Gammaproteobacteria*, and this taxon was significantly more abundant in sea ice as compared to seawater. A symbiotic relationship was proposed to exist between algae and bacteria by production of vitamin B12^29^, and algae exuded exopolymeric substances^30^,^31^, acting as osmo- and cryoprotectants, probably allowing for the survival of bacteria that are not necessarily cold-adapted^32^,^33^,^34^. However, in our metagenomic dataset, the relative abundance of genes related to the biosynthesis of vitamin B12 was significantly higher in the seawater, while the relative abundance of genes related to osmoregulation varied greatly from sample to sample, with no significant differences between sea ice and seawater.

The mean values and range for the physico-chemical indicators are similar to values previously reported for surface water in the Northwest Passage^35^. Among these indicators, salinity and nutrients were shown to be correlated with the abundance of various microbial taxa, even though variability in environmental parameters was small^35^. Interestingly, in the present study, the number of sea ice OTUs and functions that had significant negative correlations with environmental parameters was much higher than the number of OTUs and functions having significantly positive correlations. In the seawater datasets, the number of positive and negative significant correlations was more balanced, suggesting different ecological strategies or susceptibilities to environmental drivers between the microbial communities inhabiting seawater and overlying sea ice.

Most results showed higher variability in microbial communities in sea ice samples as compared to seawater. Sea ice is highly variable in terms of physiochemical properties, especially within its microenvironments^36^, as well as snow cover depth, resulting in an uneven distribution (e.g. abundance and species composition) of algae and other microorganisms at the ice bottom^37^,^38^. Accordingly, we observed variability in physical and chemical characteristics of sea ice samples, which resulted in higher variability in microbial communities when compared to the more homogeneous seawater samples. Still, the water mass in the Canadian Arctic archipelago is quite variable regarding dynamics and nutrient content^39^.

Although previous studies used different PCR primers^7^,^40^, sampled multi-year ice as compared to first-year ice here^12^,^33^,^41^,^42^ and sampled later in summer as compared to our springtime sampling in the midst of the algal bloom^11^, the community composition of the surface water and bottom layer of the sea ice samples of this study were comparable to previous studies of Arctic and Antarctic sea ice and surface water. The *Gammaproteobacteria* and *Bacteroidetes* were abundant in sea ice samples, similar to multi-year ice sampled near the geographic North Pole^12^ and in first-year ice from a Norwegian Fjord study^43^. Other taxa that were present, but at lower abundances, were *Actinobacteria, Alpha*- and *Betaproteobacteria*, which are also common to other first-year and multi-year sea ice samples^10^. As in the sea ice, both *Proteobacteria* and *Bacteroidetes* were relatively abundant in seawater samples, similar to previous reports from Antarctic and Arctic seawater^8^,^12^,^35^,^44^.

In conclusion, our study has shown that the microbial communities and their associated functional genes present around Cornwallis Island in the Canadian high Arctic are very different between sea ice and seawater, even though they were quite variable between sampling sites. The functional differences observed could be at the root of the different capacities of sea ice and seawater to degrade hydrocarbon, as recently shown using samples from the same area^45^.

## Additional Information

**How to cite this article:** Yergeau, E. *et al*. Metagenomic survey of the taxonomic and functional microbial communities of seawater and sea ice from the Canadian Arctic. *Sci. Rep.* **7**, 42242; doi: 10.1038/srep42242 (2017).

**Publisher's note:** Springer Nature remains neutral with regard to jurisdictional claims in published maps and institutional affiliations.

## Supplementary Material

Supplementary Table S1

Supplementary Table S2

## Figures and Tables

**Table 1:** Physico-chemical characterization of the surface waters and sea ice bottom samples.

	Salinity	Chl *a*	DOC	TN	POC	Total Bac.	NO_2_+NO_3_	PO_4_	Si(OH)_4_	Ice thickness	Snow thickness
		(μg l^−1^)	(μM)	(μM)	(μg l^−1^)	(10^3^ cells ml^−1^)	(μmol l^−1^)	(μmol l^−1^)	(mmol l^−1^)	(cm)	(cm)
Sea water (n = 23)	32.1 ± 0.4 (31.1–32.8)	0.3 ± 0.1 (0.1–0.5)	63.6 ± 8.7 (48.5–87.3)	11.7 ± 2.1 (6.9–14.5)	33.6 ± 18.1 (19.6–106.7)	163 ± 40 (105–257)	7.8 ± 2.7 (3.5–11.1)	1.1 ± 0.2 (0.7–1.3)	10.9 ± 4.1 (4.4–17.6)	NA	NA
Bottom ice (n = 23)	11.7 ± 2.5 (5.5–14.7)	1387.8 ± 793.0 (176–2750)	2197 ± 1799 (100–7153)	139 ± 89 (17–347)	47114 ± 27317 (3763–132712)	5638 ± 2180 (948–9198)	14.9 ± 13.7 (1.4–44.1)	6.6 ± 4.7 (2.0–20.4)	5.4 ± 3.2 (1.4–12.0)	133.0 ± 19.5 (96.2–167.6)	8.3 ± 4.3 (1.1–20.6)

Values are means ± standard deviation (range). NA: not applicable.

## References

[b1] AMAP. Snow, water, ice and permafrost in the Arctic (SWIPA). (Arctic Monitoring and Assessment Programme (AMAP), Oslo, Norway, 2011).

[b2] McLaughlin F., Carmack E. C., Ingram R. G., Williams W. & Michel C. In The Global Coastal Ocean, Interdisciplinary Regional Vol. 14 *The* Sea (eds A. R. Robinson & K.H. Brink) 1211–1242 (Wiley, 2004).

[b3] Michel C. . Biodiversity of Arctic marine ecosystems and responses to climate change. Biodiversity 13, 200–214 (2012).

[b4] Stroeve J. C. . The Arctic’s rapidly shrinking sea ice cover: a research synthesis. Climatic Change 110, 1005–1027 (2012).

[b5] Arrigo K. R., van Dijken G. & Pabi S. Impact of a shrinking Arctic ice cover on marine primary production. Geophysical Research Letters 35, L19603, doi: 10.1029/2008GL035028 (2008).

[b6] Tremblay J. É. . Climate forcing multiplies biological productivity in the coastal Arctic Ocean. Geophysical Research Letters 38 (2011).

[b7] Kirchman D. L., Cottrell M. T. & Lovejoy C. The structure of bacterial communities in the western Arctic Ocean as revealed by pyrosequencing of 16S rRNA genes. Environmental Microbiology 12, 1132–1143 (2010). 20132284 10.1111/j.1462-2920.2010.02154.x

[b8] Comeau A. M., Li W. K., Tremblay J.-É., Carmack E. C. & Lovejoy C. Arctic Ocean microbial community structure before and after the 2007 record sea ice minimum. PLoS One 6, e27492 (2011). 22096583 10.1371/journal.pone.0027492 PMC3212577

[b9] Yergeau E. & Greer C. W. In Polar Microbiology: Life in a Deep Freeze (eds R. V. Miller & L.G. Whyte) 156–165 (ASM Press, 2012).

[b10] Deming J. W. In Sea ice (eds D.N. Thomas & G.S. Dieckmann) 247–282 (Wiley Blackwell, 2010).

[b11] Collins R. E., Rocap G. & Deming J. W. Persistence of bacterial and archaeal communities in sea ice through an Arctic winter. Environmental microbiology 12, 1828–1841 (2010). 20192970 10.1111/j.1462-2920.2010.02179.x PMC2916213

[b12] Bowman J. S. . Microbial community structure of Arctic multiyear sea ice and surface seawater by 454 sequencing of the 16S RNA gene. ISME J. 6, 11–20 (2011). 21716307 10.1038/ismej.2011.76 PMC3246233

[b13] Kellogg C. T. & Deming J. W. Comparison of free-living, suspended particle, and aggregate-associated bacterial and archaeal communities in the Laptev Sea. Aquatic Microbial Ecology 57, 1–18 (2009).

[b14] Parsons T., Maita Y. & Lalli C. A manual of chemical and biological methods for seawater analysis. 173 (Pergamon Press, 1989).

[b15] Belzile C., Brugel S., Nozais C., Gratton Y. & Demers S. Variations of the abundance and nucleic acid content of heterotrophic bacteria in Beaufort Shelf waters during winter and spring. Journal of Marine Systems 74, 946–956 (2008).

[b16] Michel C. & Niemi A. Field and Laboratory Methods for Biogeochemical Analyses of Sea Ice, Seawater and Particle Interceptor Trap Particles. (Fisheries and Aquatic Sciences, Central and Arctic Region, Department of Fisheries and Oceans Canada, Winnipeg, Manitoba, Canada, 2009).

[b17] Ausubel F. M. . Short protocols in molecular biology: a compendium of methods from Current protocols in molecular biology. 5th edn, (Wiley, 2002).

[b18] Sanschagrin S. & Yergeau E. Next-generation sequencing of 16S rRNA gene amplicons. J. Vis. Exp. 90, e51709 (2014). 10.3791/51709 PMC4828026 25226019

[b19] Tremblay J. . Primer and platform effects on 16S rRNA tag sequencing. Frontiers in microbiology 6, 771, doi: 10.3389/fmicb.2015.00771 (2015). 26300854 PMC4523815

[b20] Yergeau E. . Transplanting soil microbiomes leads to lasting effects on willow growth, but not on the rhizosphere microbiome. Frontiers in microbiology 6, 1436, doi: 10.3389/fmicb.2015.01436 (2015). 26733977 PMC4685055

[b21] McMurdie P. J. & Holmes S. Waste not, want not: why rarefying microbiome data is inadmissible. PLoS computational biology 10, e1003531 (2014). 24699258 10.1371/journal.pcbi.1003531 PMC3974642

[b22] Meyer F. . The metagenomics RAST server - a public resource for the automatic phylogenetic and functional analysis of metagenomes. BMC Bioinformatics 9, 386, doi: 10.1186/1471-2105-9-386 (2008). 18803844 PMC2563014

[b23] Gomez-Alvarez V., Teal T. K. & Schmidt T. M. Systematic artifacts in metagenomes from complex microbial communities. ISME J. 3, 1314–1317 (2009). 19587772 10.1038/ismej.2009.72

[b24] Cox M., Peterson D. & Biggs P. SolexaQA: At-a-glance quality assessment of Illumina second-generation sequencing data. BMC Bioinformatics 11, 485 (2011). 10.1186/1471-2105-11-485 PMC2956736 20875133

[b25] Aziz R. . The RAST Server: Rapid Annotations using Subsystems Technology. BMC Genomics 9, 75 (2008). 18261238 10.1186/1471-2164-9-75 PMC2265698

[b26] Riedel A., Michel C., Poulin M. & Lessard S. Taxonomy and Abundance of Microalgae and Protists at a First-Year Sea Ice Station near Resolute Bay, Nunavut, Spring to Early Summer. (2003).

[b27] Aslam S. N., Michel C., Niemi A. & Underwood G. J. Patterns and drivers of carbohydrate budgets in ice algal assemblages from first year Arctic sea ice. Limnology and Oceanography (2016).

[b28] Forest A. . Biogenic carbon flows through the planktonic food web of the Amundsen Gulf (Arctic Ocean): A synthesis of field measurements and inverse modeling analyses. Progress in Oceanography 91, 410–436 (2011).

[b29] Croft M. T., Lawrence A. D., Raux-Deery E., Warren M. J. & Smith A. G. Algae acquire vitamin B12 through a symbiotic relationship with bacteria. Nature 438, 90–93 (2005). 16267554 10.1038/nature04056

[b30] Riedel A., Michel C. & Gosselin M. Seasonal study of sea-ice exopolymeric substances on the Mackenzie shelf: implications for transport of sea-ice bacteria and algae. Aquatic microbial ecology 45, 195–206 (2006).

[b31] Riedel A., Michel C., Gosselin M. & LeBlanc B. Winter–spring dynamics in sea-ice carbon cycling in the coastal Arctic Ocean. Journal of Marine Systems 74, 918–932 (2008).

[b32] Junge K., Eicken H. & Deming J. W. Bacterial activity at− 2 to− 20 C in Arctic wintertime sea ice. Applied and Environmental Microbiology 70, 550–557 (2004). 14711687 10.1128/AEM.70.1.550-557.2004 PMC321258

[b33] Deming J. & Eicken H. In Planets and life: the emerging science of astrobiology (eds Sullivan W. T. & Baross J. A.) 292–312 (Cambridge University Press, 2007).

[b34] Krembs C. & Deming J. W. In Psychrophiles: from biodiversity to biotechnology (eds Margesin R., Schinner F., Marx J. C. & Gerday C.) 247–264 (Springer, 2008).

[b35] Wells L. E. & Deming J. W. Abundance of Bacteria, the Cytophaga-Flavobacterium cluster and Archaea in cold oligotrophic waters and nepheloid layers of the Northwest Passage, Canadian Archipelago. Aquatic microbial ecology 31, 19–31 (2003).

[b36] Thomas D. N., Papadimitriou S. & Michel C. In Sea Ice (eds Thomas D. N. & Dieckmann G. S.) 425–467 (Wiley-Blackwell, 2010).

[b37] Gosselin M., Legendre L., Therriault J.-C., Demers S. & Rochet M. Physical control of the horizontal patchiness of sea-ice microalgae. Marine Ecological Progress Series 29, 289–298 (1986).

[b38] Rysgaard S., Kühl M., Glud R. N. & Hansen J. W. Biomass, production and horizontal patchiness of sea ice algae in a high-Arctic fjord (Young Sound, NE Greenland). Marine Ecology Progress Series 223, 15–26 (2001).

[b39] Michel C. . Arctic Ocean outflow shelves in the changing Arctic: A review and perspectives. Progress in Oceanography 139, 66–88 (2015).

[b40] Galand P. E., Casamayor E. O., Kirchman D. L. & Lovejoy C. Ecology of the rare microbial biosphere of the Arctic Ocean. Proceedings of the National Academy of Sciences of the United States of America 106, 22427–22432, doi: 10.1073/pnas.0908284106 (2009). 20018741 PMC2796907

[b41] Brown M. V. & Bowman J. P. A molecular phylogenetic survey of sea‐ice microbial communities (SIMCO). FEMS Microbiology Ecology 35, 267–275 (2001). 11311437 10.1111/j.1574-6941.2001.tb00812.x

[b42] Brinkmeyer R. . Diversity and structure of bacterial communities in Arctic versus Antarctic pack ice. Applied and Environmental Microbiology 69, 6610–6619 (2003). 14602620 10.1128/AEM.69.11.6610-6619.2003 PMC262250

[b43] Brakstad O. G., Nonstad I., Faksness L.-G. & Brandvik P. J. Responses of microbial communities in Arctic sea ice after contamination by crude petroleum oil. Microbial ecology 55, 540–552 (2008). 17805918 10.1007/s00248-007-9299-x

[b44] Gentile G. . Study of bacterial communities in Antarctic coastal waters by a combination of 16S rRNA and 16S rDNA sequencing. Environmental Microbiology 8, 2150–2161 (2006). 17107556 10.1111/j.1462-2920.2006.01097.x

[b45] Garneau M.-È. . Hydrocarbon biodegradation by Arctic sea-ice and sub-ice microbial communities during microcosm experiments, Northwest Passage (Nunavut, Canada). FEMS Microbiology Ecology 92, fiw130 (2016). 27387912 10.1093/femsec/fiw130

